# The Gendered Family Process Model: An Integrative Framework of Gender in the Family

**DOI:** 10.1007/s10508-018-1185-8

**Published:** 2018-03-16

**Authors:** Joyce J. Endendijk, Marleen G. Groeneveld, Judi Mesman

**Affiliations:** 1 Centre for Child and Family Studies, Leiden University, P.O. Box 9555, 2300 RB Leiden, The Netherlands; 2 Child and Adolescent Studies, Utrecht University, P.O. Box 80140, 3508 TC Utrecht, The Netherlands

**Keywords:** Gender identity, Family process, Gender stereotypes, Gender socialization, Gender development

## Abstract

This article reviews and integrates research on gender-related biological, cognitive, and social processes that take place in or between family members, resulting in a newly developed gendered family process (GFP) model. The GFP model serves as a guiding framework for research on gender in the family context, calling for the integration of biological, social, and cognitive factors. Biological factors in the model are prenatal, postnatal, and pubertal androgen levels of children and parents, and genetic effects on parent and child gendered behavior. Social factors are family sex composition (i.e., parent sex, sexual orientation, marriage status, sibling sex composition) and parental gender socialization, such as modeling, gender-differentiated parenting, and gender talk. Cognitive factors are implicit and explicit gender-role cognitions of parents and children. Our review and the GFP model confirm that gender is an important organizer of family processes, but also highlight that much is still unclear about the mechanisms underlying gender-related processes within the family context. Therefore, we stress the need for (1) longitudinal studies that take into account the complex bidirectional relationship between parent and child gendered behavior and cognitions, in which within-family comparisons (comparing behavior of parents toward a boy and a girl in the same family) are made instead of between-family comparisons (comparing parenting between all-boy families and all-girl families, or between mixed-gender families and same-gender families), (2) experimental studies on the influence of testosterone on human gender development, (3) studies examining the interplay between biology with gender socialization and gender-role cognitions in humans.

## Introduction

Gender is one of the most important organizers of social life (Blakemore, Berenbaum, & Liben, 2009), from the cradle to the grave. It shapes a large part of children’s identity development, the way they view the world, and influences the way they are talked to, the way they are parented, the opportunities they are provided with, and people’s reactions to certain behaviors, hobbies, interests, and play styles. As a consequence of these processes, boys and girls differ, for example, in toy, occupational, and activity preferences throughout childhood and adolescence (e.g., Berenbaum, Martin, Hanish, Briggs, & Fabes, 2007; Konrad et al., 2000; McHale, Kim, Dotterer, Crouter, & Booth, 2009). Also, boys are more likely to express externalizing emotions and behaviors (e.g., aggression), whereas girls are more likely to show internalizing emotions and behaviors (e.g., depression) (for meta-analyses and reviews, see Archer, 2004; Chaplin & Aldao, 2013; Hyde, Mezulis, & Abrahamson, 2008). Children’s gender development can be studied in different contexts, such as the family context, the school context, the peer group, and the larger societal context (Blakemore et al., 2009). The current article reviews and integrates research on gender development of children and adolescents in the family context. The family context is crucial for gender development, providing the first gender-related experiences that children incorporate in their gender concepts (Bem, 1981), which in turn shape the influence of other socializing agents. Also, parents play a role in some of the biological factors (genes, prenatal testosterone) associated with child gender development (e.g., Caramaschi, Booij, Petitclerc, Boivin, & Tremblay, 2012).

Several general and broad theories of child or gender development have been applied to gender socialization processes in the family context (e.g., evolutionary theories; Trivers, 1972; social role theory; Eagly, Wood, & Diekman, 2000; social learning theories; Bandura & Walters, 1963; Bussey & Bandura, 1999). However, these theories do not specifically address gender-related family processes, i.e., all gender-related biological, cognitive, and social processes that take place in or between members of the family (Whitechurch & Constantine, 1993). For example, evolutionary theories predict that different behaviors are adaptive for males and females, but are applied primarily to gender differences in aggression and parental investment. Social role theory attributes differences in behavior of women and men to the contrasting distributions of men and women into social roles, and primarily tries to explain (bio)social gendered processes not limited to the family context (Wood & Eagly, 2012). Social learning theory predicts the development of social behaviors from processes such as observational learning and reinforcement/punishment from social agents in the child’s environment. There are also some family context frameworks or models that mainly focus on very specific gender-related aspects or processes in the family system, such as children’s gender cognitions (e.g., gender-schema theories; Bem, 1981, 1983; Martin & Halverson, 1981, 1987), or integrate biological and environmental factors in a family system, but are not focused on gender (e.g., genotype–environment effects; Scarr & McCartney, 1983).

Previous reviews provided valuable overviews of biological, social, or cognitive perspectives on children’s gender development in the family context, but did not integrate these different perspectives (see Blakemore et al., 2009; Eccles, Freedman-Doan, Frome, Jacobs, & Yoon, 2000; Maccoby & Jacklin, 1974; McHale, Crouter, & Whiteman, 2003). Several gender development scholars have signaled the need for a comprehensive explanatory model combining biological, social, and cognitive perspectives on gender development (e.g., Berenbaum, Martin, & Ruble, 2008; Berenbaum, Blakemore, & Beltz, 2011; Leaper, 2015). Such a model would be essential for the continuation and expansion of the study of gender in the family context and for the understanding of child gender development. Therefore, in the current article, we first review previous research on gendered family processes. From this review, pathways between gender-related biological, social, and cognitive factors at both the parent and child level were identified and integrated in a new integrative research framework: the gendered family process model (GFP model).

**Fig. 1:**
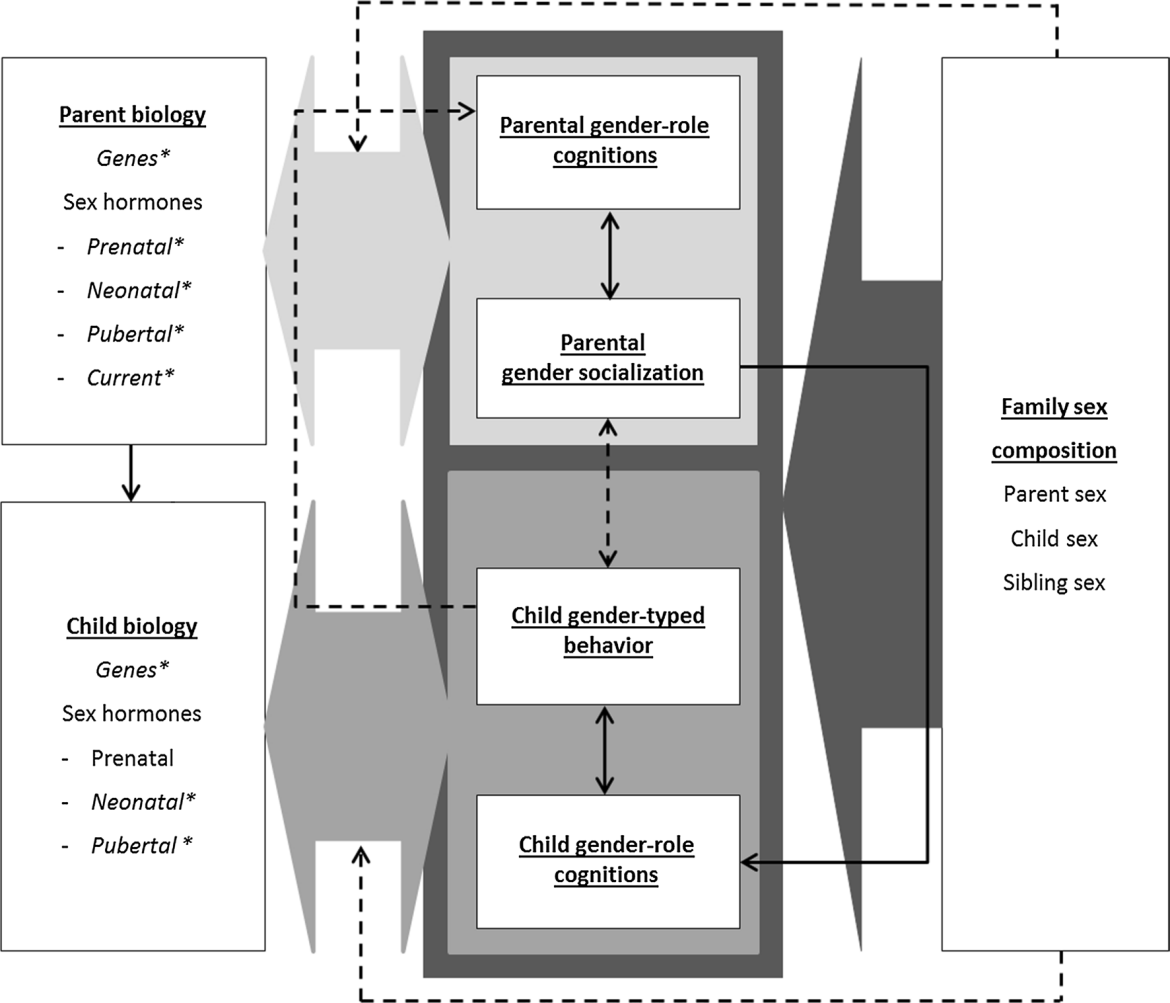
The gendered family process model. *Note* Large gray arrows refer to direct associations with the constructs it points to, and to moderation of the associations between the constructs it points to. Dashed arrows and constructs and associations in italics marked with a * represent areas and associations for which future research is necessary, because of inconsistent evidence, too little empirical evidence, or theoretical support only

The GFP model (Fig. 1, see Table 1 for construct definitions) is based on family system theories (e.g., Whitechurch & Constantine, 1993), biosocial perspectives on the family (e.g., Troost & Filsinger, 1993), and more specific biological, social, and cognitive theories about gender development (e.g., hormonal perspectives, behavioral-genetic perspectives, social learning theory, gender-schema theories). In family system theories and biosocial family theories, the family is viewed as a system encompassing biological, social, and cognitive processes. Understanding of gender-related family processes requires considering the family as a whole rather than as “conglomerates of separate individuals” (Whitechurch & Constantine, 1993, p. 340), and attention to both biological and social or psychological factors. Thus, an adequate framework should take into account all members of the family (family composition) and the ways in which they interact with or influence each other (i.e., family processes). The family processes can be at a dyadic level (parent–child), triadic level (parent–child–sibling; mother–father–child), or family level (all family members). With a family systems approach, it is possible to connect different theoretical perspectives on gender development that complement each other, especially when we view biological, social, and cognitive factors as subsystems within each family member, that together comprise the larger family system (Minuchin, 1974). The model and associated evidence are described below in separate domains—biological, social, cognitive—and integrations between the domains are provided throughout the article.

**Table 1:** Definitions of the constructs used in the gendered family process model

Construct	Definition
Parental gender socialization	All ways (intentional and unintentional) in which parents teach their children about the social expectations and attitudes associated with gender (Henslin, 2001); e.g., modeling, differential treatment of boys and girls, talking about gender
Gender roles	Societal norms regarding appropriate roles and behaviors for men, women, boys, and girls (Eagly et al., 2000)
Gender-typed behavior	All aspects of behavior that show gender differences; e.g.,
	Toy, occupational, and activity preferences (e.g., Berenbaum, Martin, Hanish, Briggs, & Fabes, 2007; Konrad, Ritchie, Lieb, & Corrigal, 2000)
	Social-emotional behavior such as internalizing and externalizing behavior problems (e.g., Archer, 2004; Chaplin & Aldao, 2013)
	Academic achievements (e.g., Else-Quest, Hyde, & Linn, 2010)
Gender-role cognitions	Cognitions related to gender; e.g., awareness of gender categories, understanding of gender constancy, (knowledge of) gender stereotypes, intergroup attitudes, gender identity aspects (Halim & Ruble, 2010), gender cognitions that influence social information processing (Crick & Dodge, 1994)
Family sex composition	Sibling sex configuration (same-gender siblings, mixed-gender siblings) and parent sex configuration (e.g., single-parent family, two-parent family, heterosexual, homosexual)

To restrict the complexity of the model, the focus is only on proximal processes within the family context. However, according to the ecological systems model (Bronfenbrenner, 1979) the family system is nested in and influenced by the larger societal and cultural environment. Important distal factors influencing gender-related processes in the family context that are not covered in the current model are among others gender socialization by peers (for a review, see Rose & Rudolph, 2006), societal gender roles, the level of gender equality in a society, and the gender-related values and practices of a culture (i.e., gender as a social construction: Baxter & Kane, 1995; Charles & Bradley, 2009; Manago, Greenfield, Kim, & Ward, 2014; Williams & Best, 1990; Yu & Lee, 2013). Other important distal factors are the evolutionary processes behind the development of differentiated gender roles, such as parental investment and sexual selection (Trivers, 1972). These evolutionary processes are not included in the model, because the evolutionary perspective generally does not yield predictions that can be tested empirically (e.g., Blakemore et al., 2009).

## Biological Perspectives: The Role of Parent and Child Biology in Family Process

### Children’s Biological Characteristics and Gender-Typed Behavior (Fig. 2)

The model includes an association between child biology and child behavior, for which a direction from child biology to child behavior seems most likely. There is ample evidence that especially the child’s testosterone (T) levels have “organizational” and “activational” effects on the child’s gender-typed behavior as reviewed by Hines et al. (2015, 2016b). Organizational effects of T are thought to be the more permanent effects of T on brain structures and related behaviors, whereas activational effects are the more temporary alterations of brain functioning and behavior related to circulating levels of hormones (Berenbaum & Beltz, 2011). Decades of research have demonstrated that girls who are exposed to high levels of T prenatally, due to the genetic disorder congenital adrenal hyperplasia (CAH), show masculinization of several behaviors, including male-typical play and interests, reduced female-typical play and interests, better spatial ability, and more aggression and substance misuse (for reviews, see Berenbaum & Beltz, 2016; Hines, Constantinescu, & Spencer, 2015). It should be mentioned that the most consistent effects have been found for gender-typed play and interests, but less consistently for sex-typed psychosocial problems, gender identity, and spatial abilities.

**Fig. 2:**
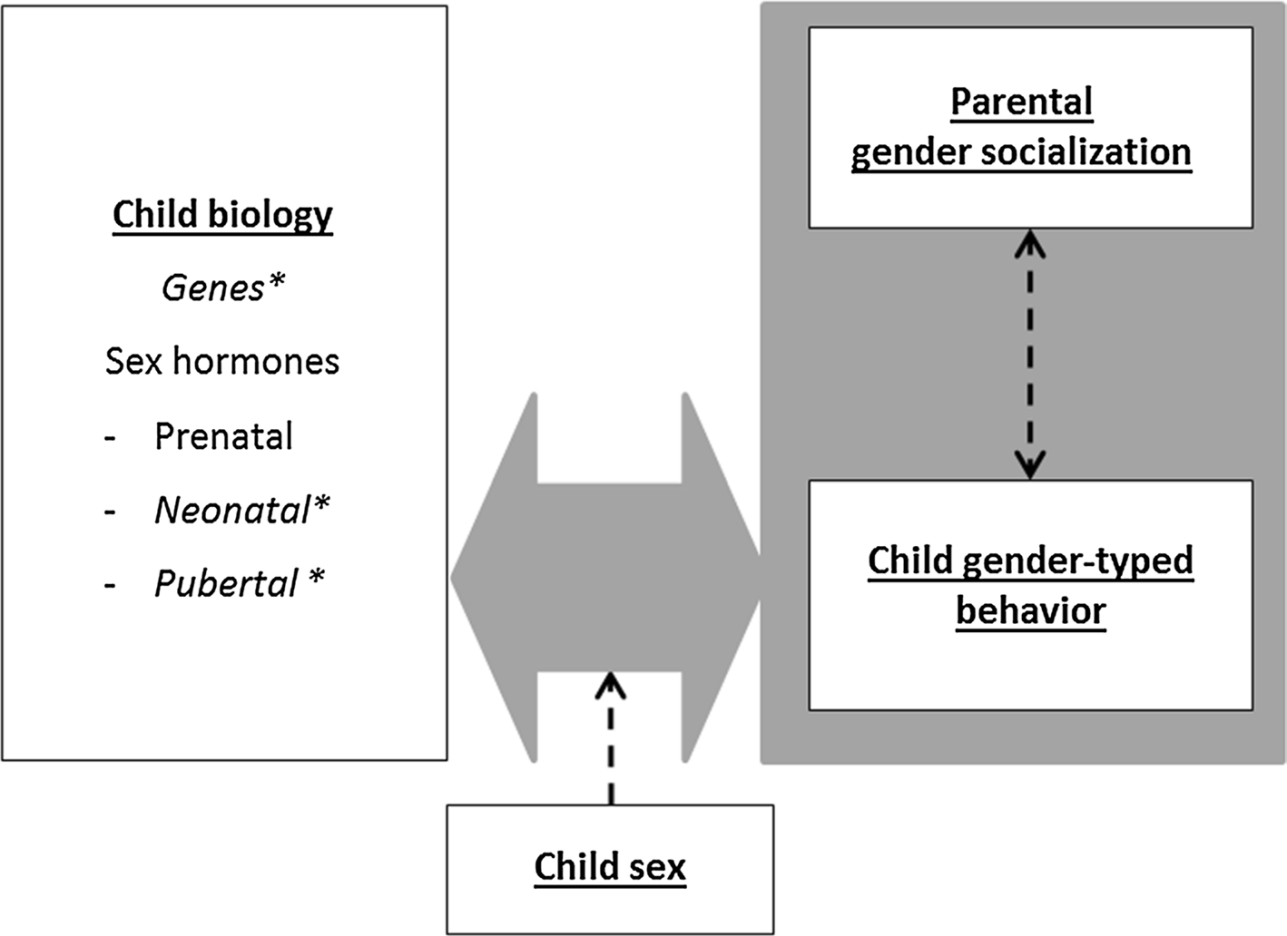
Children’s biological characteristics and gender-typed behavior

A small number of studies have linked natural variations in prenatal T levels, measured in maternal blood during pregnancy or amniotic fluid, to variations in both girls’ and boys’ gender-role behavior (Auyeung et al., 2009; for a review, see Cohen-Bendahan, Van de Beek, & Berenbaum, 2005). There are also studies that do not find an association between natural variations in prenatal T and gender-role behavior (Knickmeyer et al., 2005; Van de Beek, van Goozen, Buitelaar, & Cohen-Kettenis, 2009). Much more studies have used the ratio of the second to the fourth digit of the hand, on the assumption that this digit ratio reflects prenatal androgen exposure. These studies show inconsistent associations with many aspects of gender-typed behavior, supposedly because the relationship of finger ratios to prenatal androgen exposure appears to be too weak to be useful in studies of typically developing individuals (Hines et al., 2015). However, there is compelling evidence of a link between digit ratio and sexual orientation in women, with digit ratios of homosexual women being smaller than those of heterosexual women (Breedlove, 2017).

Not only prenatal T levels are implicated in the child’s gender development. Also, early postnatal T, which is highest in male infants in the first 6 months of life, has been associated in several studies with reduced language development in both boys and girls, gender-typical play in boys, and gender-atypical play in girls, but not with autistics traits (for a review, see Hines et al., 2016b). The rise of androgen or estrogen levels during puberty also has important organizational and activational effects on the adolescent’s brain and behavior (Berenbaum & Beltz, 2011; Peper, Hulshoff, Pol, Crone, & Van Honk, 2011). In an extensive review of the literature, Berenbaum and Beltz (2011) only found evidence of long-lasting organizational effects of circulating T levels during puberty on gender identity and not on behavior. The review also described some evidence that the rise in estrogens during puberty is linked to gender-typical behavior problems that generally emerge during adolescence such as depression, eating disorders, and anxiety disorders. However, it is unclear whether these effects are organizational or activational. Another mini-review of neuroimaging studies concluded that the changes in sex steroids (androgens and estrogens) during puberty are involved in structural reorganization of gray and white matter in the brain (Peper et al., 2011). However, several of these sex differences in brain structure are already visible in 1-month-old infants (Dean et al., 2018). With regard to activational effects of T on gender-typed behaviors, the rise in T levels during puberty in boys has been associated with increased risk taking (Vermeersch, T’Sjoen, Kaufman, & Vincke, 2008), sensation seeking, and sensitivity for reward (Forbes et al., 2010), but not with increased aggression (for a review, see Archer, 2006). In addition, administration of T in adults has been related to various aspects of social–emotional behavior, such as better emotion detection for threatening faces, reduction of fear, increased motivation to act, and increased sensitivity for reward (for a review, see Bos, Panksepp, Bluthé, & van Honk, 2012).

In addition to the classic, and dominant, focus on the influence of sex hormones in the field of gender development, there is an emerging view that direct genetic effects play an important role as well (Ngun, Ghahramani, Sánchez, Bocklandt, & Vilain, 2011). Genetic effects on gender development are difficult to investigate, but evidence is starting to emerge indicating that genes on both the X and Y chromosome are associated with behavioral gender differences (for a review, see Arnold, 1996, 2009; Ngun et al., 2011). For example, manipulated mice that are genetically male, but hormonally female, due to deletion of *Sry* gene on Y chromosome responsible for testis formation, show aggression and parenting behaviors like pup retrieval at the level of normal male mice (Gatewood et al., 2006). Other studies using the same mouse model have identified additional behavioral gender differences that can be attributed to genes on the sex chromosomes, including habit formation in a food reinforcement paradigm, pain sensitivity, and affiliative and asocial behavior toward intruders (see Arnold, 2009). In addition, studies of genetically manipulated female mice that did not differ hormonally found increased anxiety in female mice with one X chromosome compared to female mice with two X chromosomes, indicating X gene(s) to be involved in modulating fear reactivity (Cox, Bonthuis, & Rissman, 2014). Together, these studies indicate that genes on the sex chromosomes have an independent effect on gender differences in behavior, when controlling for the effects of androgens.

There are humans with chromosomal abnormalities similar to these mice. Research from males with Klinefelter syndrome (extra X chromosome) has found that these men show impaired social processing, verbal abilities, and cognitive functioning compared to normal controls (Cox et al., 2014). Girls with Turner syndrome (absence of or abnormality in one X chromosome) have been found to be at higher risk of autism and have impaired visuospatial skills, memory, and attention (Cox et al., 2014). Thus, there is also evidence from studies with humans for effects of sex-linked genes on the X chromosome on behaviors that show normative gender differences. However, some of the effects might be confounded by hormonal abnormalities associated with Klinefelter (lower T during puberty; Wosnitzer & Paduch, 2013) and Turner syndrome (sex hormone insufficiency; Trolle, Hjerrild, Cleemann, Mortensen, & Gravholt, 2012) as few studies have sorted out effects of hormones on behavior in these patients (Cox et al., 2014).

Very few studies have examined possible moderators of the association between child biological factors and gender-typed behavior. First, sex might be a moderator, because studies in nonhuman species suggest more behavioral effects of high prenatal T associated with CAH in females than in males, even though both males and females can be affected by CAH (Berenbaum & Beltz, 2011). One study confirmed this moderation by child sex in humans, demonstrating an association between prenatal T variability and gender-role behavior in girls only (i.e., higher prenatal T associated with more gender-atypical behavior) (Hines et al., 2002). These findings might indicate that a hormonal predisposition toward cross-gendered behavior might be counteracted more by parental socialization influences in boys than in girls (Hines et al., 2002). However, more research is necessary to determine whether T has stronger effects on females or males.

Second, there is some evidence for an interaction between parent (gender) socialization and the child’s T levels. Money and Ehrhardt (1972) were among the first researchers interested in the interplay between gonadal hormones and environmental factors in human gender development. They theorized that the differential exposure of boys and girls to gonadal hormones in the womb was related to subtle gender differences in brain development and behavior, which together with socialization influences would play a critical role in gender development. We only know of two studies demonstrating that child T and parental socialization together determine child gender behavior (Booth, Johnson, Granger, Crouter, & McHale, 2003; Udry, 2000). Booth et al. showed that when parent–child relationship quality was high, the association between T and risk-taking behavior or depressive symptoms was less strong than when parent–child relationship quality was low. In addition, Udry demonstrated that for women with low prenatal exposure to T, their mothers’ encouragement of femininity had a strong effect on gendered behavior in adulthood, whereas for women with high prenatal T exposure, their mothers’ gender socialization had no influence.

### Child Biology, Child Gender-Typed Behavior, Parent Gender Socialization (Fig. 2)

Biological characteristics of children might also indirectly influence parental gender socialization via child gender-typed behavior. Research on disorders of sex development (DSD) (e.g., CAH, girls with complete androgen insensitivity syndrome who are genetically male but do not have effective androgen exposure, boys without a penis who are reared as girls) could provide some evidence in this regard. Children with DSDs provide us with the opportunity to examine whether their hormonally/genetically induced gender-atypical behavior leads to differential treatment by parents and enable us to disentangle effects of genetic sex versus early hormonal exposure on parental socialization. However, the results of the few studies that have been conducted are mixed. Some studies found that parents did not treat their daughters with CAH differently than they treated their unaffected daughters (for a review, see Cohen-Bendahan et al., 2005). One study found that both mothers and fathers were observed to encourage girl-typical toy play more in their daughters with CAH than in their unaffected daughters (i.e., counteract girls’ natural predispositions; Pasterski et al., 2005), whereas another study showed that parents reported to encourage more boy-typical and less girl-typical toy play in girls with CAH compared to unaffected girls, which partially mediated the association between CAH status and gender-typical toy play (i.e., reinforce girls’ natural predispositions; Wong, Pasterski, Hindmarsh, Geffner, & Hines, 2013). These different findings might be due to methodological differences between the studies. Those finding no significant differences in socialization used single items to assess parental encouragement or did not focus on socialization of specific behaviors. The studies that did find differences, but in opposite directions, used either parent reports or structured observations, which provide a different type of information. Questionnaires can assess a broad range of naturalistic behaviors, but might be hampered by subjectivity, whereas observations, albeit more objective, only focus on specific behaviors in a structured setting with an experimenter present. Evidence for the idea that hormonally induced gender-atypical behavior in girls with CAH influences parental socialization and not the other way around, comes from observational studies showing that CAH girls did not play more with boys’ toys when a parent was present than when they played alone (Pasterski et al., 2005).

Evidence for the pathway in which genetically or hormonally predisposed differences in behavior or temperament of boys and girls might evoke differential parental reactions can also be found in studies on evocative gene–environment correlation (rGE; Plomin, DeFries, & Loehlin, 1977; Scarr & McCartney, 1983). Evocative rGE refers to the evocative effect that genetically predisposed child characteristics have on parent behavior. There is a large body of research, mostly using self-report data, that suggests genetic child-driven evocative effects on parenting (see for meta-analytic evidence Klahr & Burt, 2014). Large population-based longitudinal twin studies have shown that children with a cooperative and/or prosocial predisposition are more likely to elicit positive reactions from their mothers and fathers, whereas children with tendencies toward disruptive behavior elicit negative reactions from their mothers and fathers (e.g., Boeldt et al., 2012; Jaffee et al., 2004; Larsson et al., 2008). Also, several adoption studies found that adopted children with a genetic predisposition toward antisocial behavior (from their biological parents) evoked more harsh and inconsistent discipline from their adoptive mothers and fathers (e.g., Ge et al., 1996; Riggins-Caspers, Cadoret, Knutson, & Langbehn, 2003). It should be mentioned that the effects in these studies were modest. With the results from these studies in mind, one can argue that hormonally or genetically induced differences in behavior of boys and girls elicit differential treatment by parents, which, in turn, might enhance the biologically predisposed gender differences in children’s behavior.

### Parent Biology and Child Biology (Fig. 3)

The GFP model includes a direct path from parent-to-child biology, because parents and children are genetically related. In addition, there is ample evidence that children’s T levels are for a large part genetically determined (Caramaschi et al., 2012; Harris, Vernon, & Boomsma, 1998; Hoekstra, Bartels, & Boomsma, 2006). Heritability estimates ranged from 66 to 85% (Harris et al., 1998; Meikle, Stringham, Bishop, & West, 1988) for adolescent males and 41 to 52% for adolescent females (Harris et al., 1998; Hoekstra et al., 2006). Nonshared environmental influences explained the rest of the variance (Harris et al., 1998; Hoekstra et al., 2006). When measures were corrected for daily fluctuations in T levels and measurement error, the variance in T levels would be practically entirely explained by genetic effects (Hoekstra et al., 2006). In infancy, variation in T levels was entirely explained by shared environmental factors (57%), such as maternal hormone levels, maternal smoking behavior, and diet during pregnancy, and nonshared environmental factors (43%), such as position in the womb or differential parenting practices (Caramaschi et al., 2012).

**Fig. 3:**
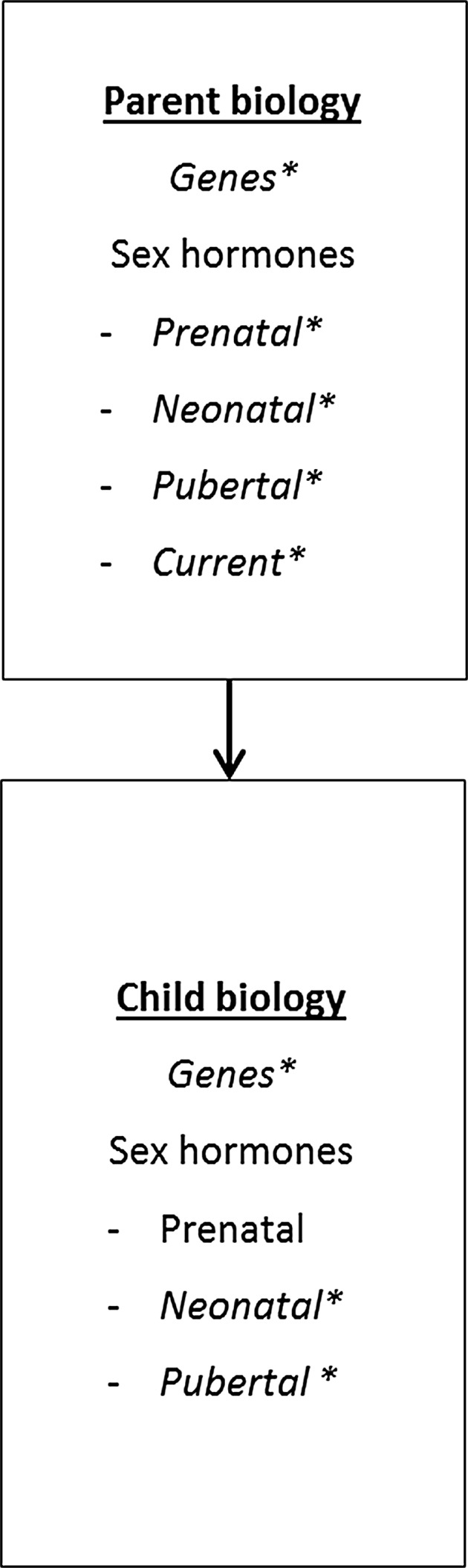
Parent biology and child biology

The prenatal T environment is also influenced by mothers’ circulating T levels. Studies of pregnant women with elevated androgen levels or women who used androgenic hormones during pregnancy show that T can pass from the maternal system to the fetus as indicated by higher fetal T levels (Barbieri, 1999; Ehrhardt & Money, 1967). In contrast, studies comparing mothers carrying fetuses with or without CAH or mothers carrying male or female fetuses found no significant differences in maternal T levels between the groups, indicating that T does not appear to pass from the fetus to the mother (Hines et al., 2002; Meulenberg & Hofman, 1991).

### Parents’ Biological Factors and Gender Socialization (Fig. 4)

In the GFP model, parents’ biological factors are linked to gender socialization, because higher circulating T levels in men compared to women are associated with lower parental involvement in fathers compared to mothers (Gettler, McDade, Feranil, & Kuzawa, 2011; Kuzawa, Gettler, Huang, & McDade, 2010; van Anders, Tolman, & Volling, 2012; Wingfield, Hegner, Dufty, & Ball, 1990). In the parenting context, the influence of T is often presented within a trade-off framework that contrasts low T levels and parental involvement with high T levels and competitive challenges or mating (van Anders et al., 2012). According to the “challenge hypothesis,” the association between T and parenting is reciprocal, with high T levels inhibiting parenting, whereas cues associated with children, child care, or parenting decrease T levels in both mothers and fathers (Gettler et al., 2011; Kuzawa et al., 2010; Wingfield et al., 1990).

**Fig. 4:**
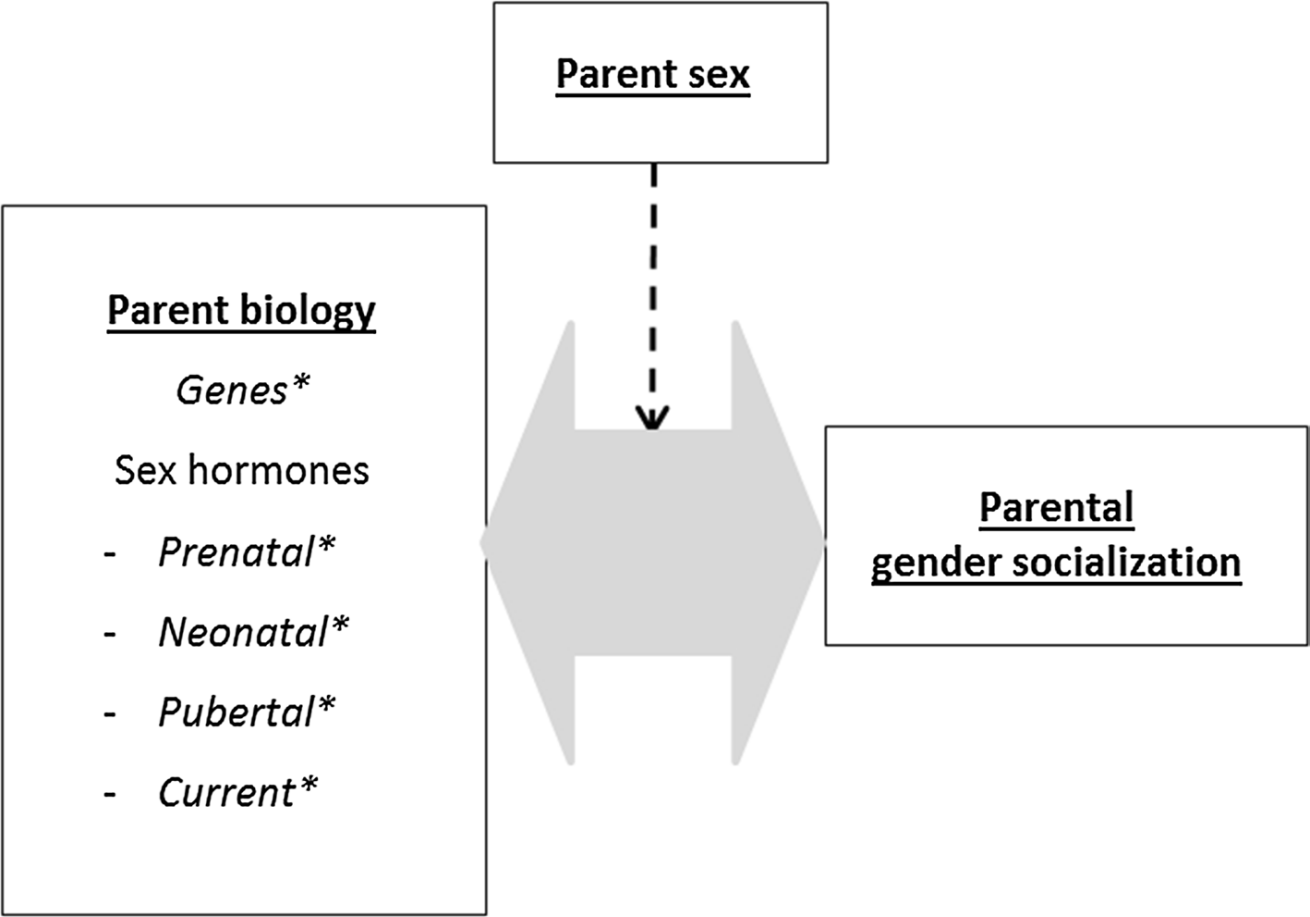
Parents’ biological characteristics and gender socialization

Mothers’ and fathers’ basal T levels might not only be related to parental involvement, but also specifically to gender socialization practices (Cohen-Bendahan et al., 2005). The same might be true for parents’ prenatal, early postnatal, or pubertal T levels. For example, mothers with high T levels (current, prenatal, postnatal, pubertal) may parent their daughters differently than mothers with low T levels, possibly because they have opposite-sex interests or reinforce their daughters’ male-typical behavior.

### Biological Factors and Gender-Role Cognitions (Fig. 5)

The GFP model includes associations between biological factors and gender-role cognitions for both parent and child, as there is accumulating evidence that gender cognitions have a neurobiological basis (for a review, see Amodio, 2014). More specifically, neuroimaging studies found that individual differences in gender stereotypes were associated with differential activity in the brain during social judgment tasks, and especially in regions linked to semantic retrieval and categorization (Mitchell, Ames, Jenkins, & Banaji, 2009), regions frequently linked to social cognition (Contreras, Banaji, & Mitchell, 2012), and areas associated with evaluative processing and the representation of action knowledge (Quadflieg et al., 2009). However, these studies have been conducted in adults and results still need to be confirmed in children.

**Fig. 5:**
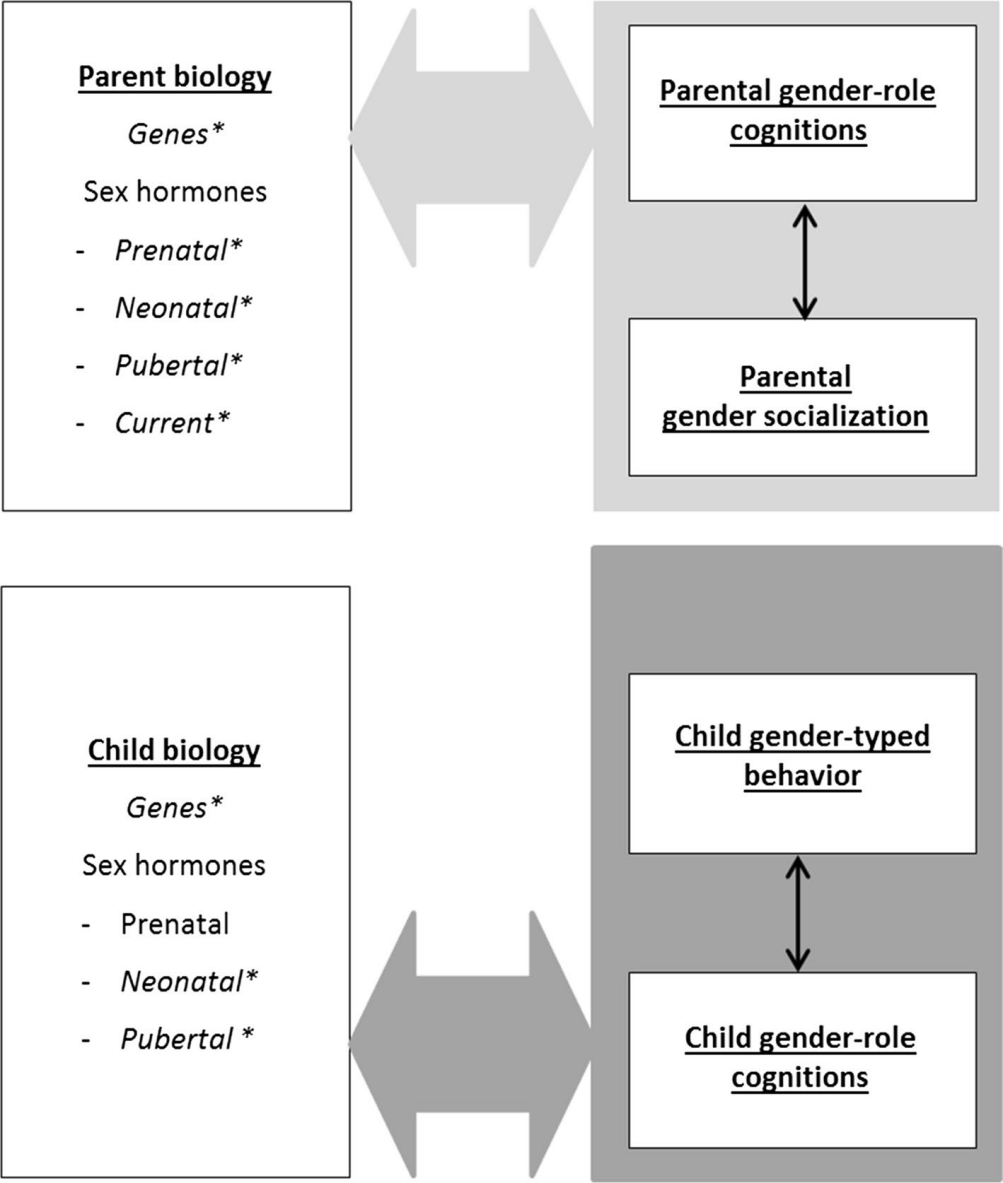
Biological factors and gender-role cognitions

There are also some studies that examined the interplay between biological factors and gender-role cognitions in adults, showing that T and gender stereotypes have an interactive effect on gender differences in cognition (Hausmann, Schoofs, Rosenthal, & Jordan, 2009) and math performance (Josephs, Newman, Brown, & Beer, 2003). These studies found that T levels only influenced math or cognitive performance when gender stereotypes were activated, but the direction of effects was different for men and women and dependent on the task at hand. For example, when gender stereotypes were activated in the math study, high T males performed better than low T males, whereas low T females performed better than high T females (Josephs et al., 2003). These results indicate that, when stereotypes are activated, men with naturally high T levels use math tests as a way to maintain their high status in math, while high T women see math tests as a threat to their status.

It also seems plausible that T levels in parents and children may have an indirect influence on their gender cognitions. According to gender-schema theories, behavior may modify gender cognitions; for example, a girl with biologically induced male-typed activity interests (e.g., football) may expand her stereotypes about what is appropriate behavior for boys and girls (e.g., “boys and girls like to play football”), to restore congruence between her stereotypes and behavior (Liben & Bigler, 2002; Martin & Dinella, 2012). However, one study that examined this hypothesis in girls with CAH found no significant association between CAH status and gender-role stereotypes (Endendijk, Beltz, McHale, Bryk, & Berenbaum, 2016). Two other studies in girls with CAH have found associations between prenatal T levels and two other types of gender cognitions: self-socialization of gender-typical behavior and gender identity. More specifically, girls with CAH have been found to show reduced self-socialization of gender-typical behavior (Hines et al., 2016a) and increased cross-gender identification (Pasterski et al., 2015).

## Social Approaches: The Parent–Child Relationship

### Parental Gender Socialization, Parent Gender Differences in Socialization, and Child Gender-Typed Behavior (Fig. 6)

Social learning theories provide important predictions with regard to the link between parental socialization and child gender-typed behavior as represented in the GFP model. Originating from behaviorism, social learning theories were developed in the 1960s to study the development of social behaviors (Bandura, Ross, & Ross, 1961; Bandura & Walters, 1963). Mischel (1966) was the first to apply social learning principles to children’s gender development. Central to these theories are the concepts of imitation/modeling and reinforcement/punishment.

**Fig. 6:**
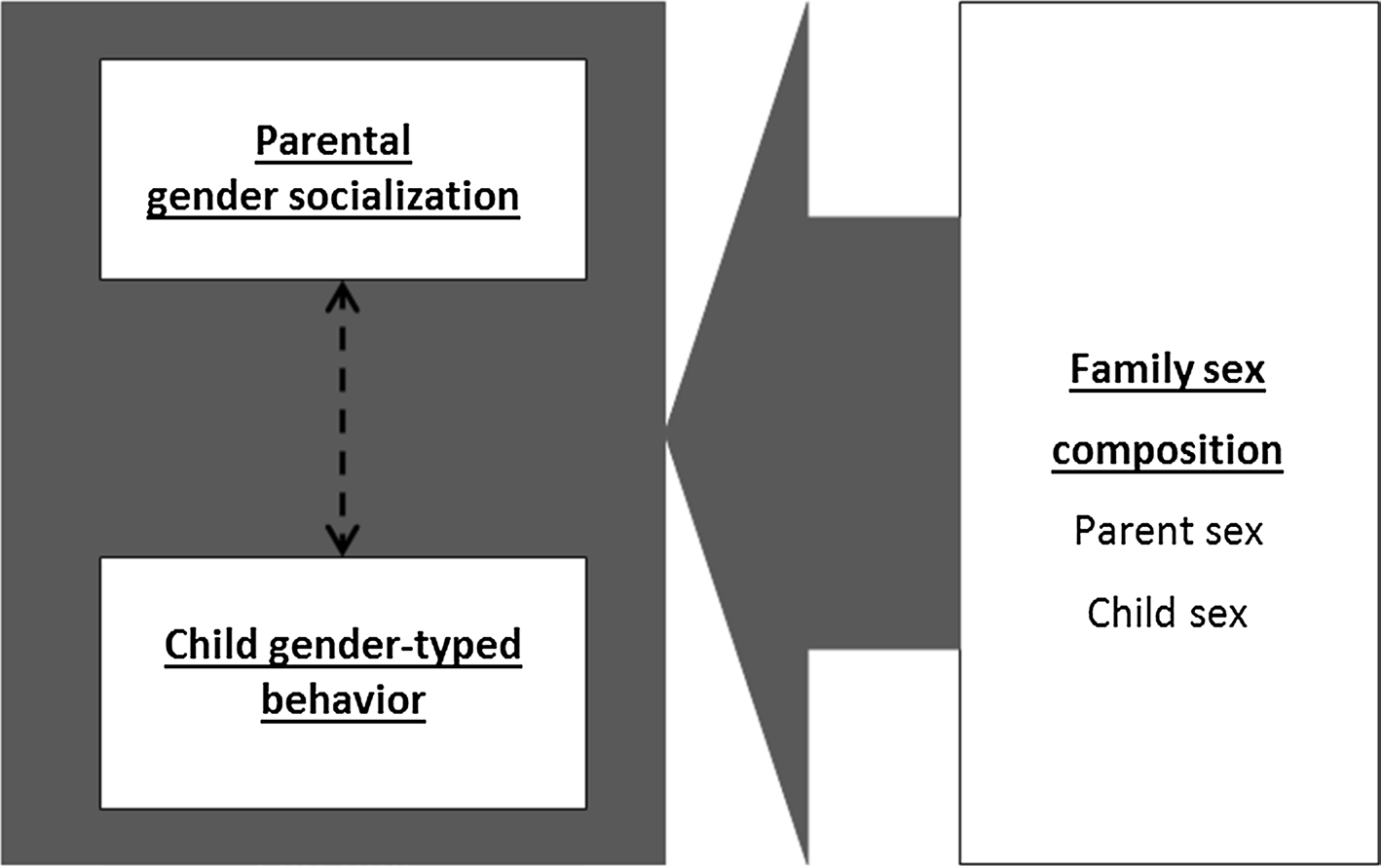
Parental gender socialization and child gender-typed behavior

Observational learning from available models, especially same-gender models, in the child’s environment is an important factor in children’s gender development. In the family context, much gender-related information is available for the child to observe and imitate. Parents are, for example, models for gender-typical behavior through their own behaviors, occupations, and interests. By observing the differences between mothers and fathers, children will learn how males and females act (see also section on family sex composition for modeling processes in other family types). Even though gender roles have become more egalitarian in the past decades, the traditional homemaker–breadwinner division is still visible in current-day families. Mothers are more likely to be the primary caregivers of young children (Huerta et al., 2013; The Fatherhood Institute, 2010) and perform 2–3 times more domestic work in and around the house than fathers, regardless of mothers’ employment status (Bianchi, Milkie, Sayer, & Robinson, 2000; Demo & Acock, 1993). Women are also more likely to work part-time than men, that is 57–80% of part-time workers are women in OECD countries (OECD, 2016). Further, women continue to be underrepresented in leadership positions in government and business (Eagly & Sczesny, 2009), and certain occupations remain primarily male-dominated (e.g., STEM fields, mechanical and construction trades) or female-dominated (e.g., education, care, social work; Lippa, 2010; Lippa, Preston, & Penner, 2014).

In addition, there is a large body of literature showing differences in parenting and parental involvement between mothers and fathers. For example, there is meta-analytic evidence that mothers talk more to their children than fathers do (Leaper, Anderson, & Sanders, 1998). Also, mothers have been found to be more sensitive and responsive to their children than fathers are (e.g., Barnett, Deng, Mills-Koonce, Willoughby, & Cox, 2008; Hallers-Haalboom et al., 2016; Lovas, 2005). Moreover, fathers appear to use more harsh discipline and power assertion than mothers do (e.g., Gunnoe & Mariner, 1997; Power, McGrath, Hughes, & Manire, 1994; Tulananda & Roopnarine, 2001). Last, fathers engage more in physical and rough-and-tumble play with their children, whereas mothers engage more in cognitive play and role playing (for a review, see Paquette, 2004).

Some studies find evidence for an association between the observation of parental role models and child gender-typed behavior in middle childhood and early adolescence (e.g., Crouter et al., 1995; Serbin, Powlishta, & Gulko, 1993). However, the mechanisms underlying the associations are unclear and the effect is not found in other studies with younger children (e.g., Weisner, Garnier, & Loucky, 1994). For example, a more traditional division of housework between parents was associated with more involvement with housework in girls and more involvement with male-typed chores in boys (Crouter et al., 1995) and more traditional occupational and peer preferences (Serbin et al., 1993).

A central aspect of social learning theory is the hypothesis that gender development is influenced in part by children’s tendencies to imitate same-sex models more than opposite-sex models. When we apply this hypothesis to the family context, one would expect boys to be more likely to imitate fathers, whereas girls would be more likely to imitate mothers. However, the evidence with regard to same-sex modeling of parents is inconsistent. For example, in the review by Maccoby and Jacklin (1974), it was concluded that most studies did not support same-sex modeling. In response to this conclusion, Perry and Bussey (1979) reconceptualized the way in which same-sex modeling influences gender development, by posing that “children discern what behaviors are appropriate for each sex by watching the behavior of *many* male and female models and by noticing differences between the sexes in the frequency with which certain behaviors are performed in certain situations” (p. 1700). Experimental evidence indeed supports this hypothesis, showing that children are more likely to imitate same-sex models only in situations when there was consensus within a same-sex group of models and a clear differentiation in behavior compared to a group of the other sex (Perry & Bussey, 1979). This work helps to explain why children’s gender-typed behavior was poorly related to that of the same-sex parent and indicates that parents only for a small part influence children’s gender development via modeling. However, parents do spend more time with same-sex children and adolescents than with opposite-sex children and adolescents (Crouter et al., 1995; McHale et al., 1999, 2000), leading to more possibilities for imitating the same-sex parent. More recent studies in particular show evidence of same-sex modeling of parental smoking and drinking behavior (Loureiro, Sanz-de-Galdeano, & Vuri, 2010; Vanassche, Sodermans, Matthijs, & Swicegood, 2014).

It should be mentioned that not every behavior that is observed by children is also performed. The actual performance is related to the consequences children anticipate in response to certain behaviors, from their parents or other people. This process is distinct from the modeling/imitation processes discussed above in that it focuses more on the social learning processes of reinforcement and punishment. In general, social learning theory states that children are more likely to perform behaviors again in the future when they (or others) are rewarded for it than when they (or others) are punished for these behaviors (Bandura & Walters, 1963; Mischel, 1966). Parents can reinforce/punish gender-typed behavior in children in several ways that are discussed below.

First, parents often create a highly gendered environment for their children by the toys, clothes, activities, and chores they choose for them (e.g., Crouter, Manke, & McHale, 1995; Fisher–Thompson, 1993; Pomerleau, Bolduc, Malcuit, & Cossette, 1990), the books or media they expose their children to (Birnbaum & Croll, 1984; Gooden & Gooden, 2001), and even by the names they give their children (Barry & Harper, 1995). This process is also called “channeling or shaping” children’s gender development (Blakemore et al., 2009; Eisenberg, Wolchik, Hernandes, & Pasternack, 1985). There are hardly any studies demonstrating the influence of these processes on children’s gender-typed behavior (Berenbaum et al., 2008). One study found that children’s ability to label gender was associated with mothers’ initiation of and positive reactions to gender-typical toy play (Fagot, Leinbach, & O’Boyle, 1992). There is, however, more evidence of parental channeling or shaping of children’s gendered cognitions (see section on Cognitive Approaches).

Second, parents can provide direct gender-related instruction to their children, by the way they talk to their children about gender (Gelman, Taylor, & Nguyen, 2004). To our knowledge, only four studies have systematically examined gender socialization via parent–child communication about gender (DeLoache, Cassidy, & Carpenter, 1987; Endendijk et al., 2014; Friedman, Leaper, & Bigler, 2007; Gelman et al., 2004). These studies showed that parents used gender labels (e.g., using the masculine label for gender-neutral picture-book characters playing with water guns), evaluative comments about gender-typical and atypical behavior (e.g., “Look, these girls are having fun baking cookies”), and explicit stereotypic comments (e.g., “Boys don’t play with dolls”) to highlight gender as a salient issue and to emphasize the appropriateness of certain behaviors for boys and girls. One of these studies also provided first evidence for the idea that talking about gender is an important factor in children’s gender development, by showing that there is a close correspondence between the way mothers and their children talk about gender (Gelman et al., 2004). However, Gelman et al. could not determine why mother and child gender talk were so much alike, because of the correlational design of the study in which mother and child gender talk were assessed at the same time.

Third, when parents respond differently (reward, punishment) to the same behaviors in boys and girls, or when parents use different parenting practices with boys and girls, children will learn that boys and girls are different and that certain behaviors are appropriate for boys, whereas other behaviors are appropriate for girls. Both Maccoby and Jacklin (1974) and Lytton & Romney (1991) found very little evidence for gender-differentiated parenting when they reviewed the literature on parents’ differential reactions to boys’ and girls’ behaviors, except for encouragement of sex-typed activities (Lytton & Romney, 1991). However, more recent studies do find evidence for gender-differentiated parenting. For example, parents are more likely to respond positively to girls’ than to boys’ prosocial behavior (Hastings, McShane, Parker, & Ladha, 2007), to react with increasing harsh discipline to boys’ than to girls’ difficult or noncompliant behavior (McFadyen-Ketchum, Bates, Dodge, & Pettit, 1996), punish boys more often for their aggression than girls (Eron, 1992), but when the angry and noncompliant behaviors continue they give into boys more often than to girls (Chaplin, Cole, & Zahn-Waxler, 2005; Radke-Yarrow & Kochanska, 1990). Parents have also been found to treat boys and girls differently with regard to physical care in non-Western societies or financial investments in Western societies (for a review, see Lundberg, 2005), emotion socialization (e.g., Chaplin et al., 2005; Fivush, 1998; Fivush, Brotman, Buckner, & Goodman, 2000), conversations (see meta-analysis by Leaper et al., 1998), risk taking (e.g., Morrongiello & Dawber, 1999; Morrongiello & Hogg, 2004), and play style (e.g., physical play or pretend play; Lindsey & Mize, 2001; Paquette, Carbonneau, Dubeau, Bigras, & Tremblay, 2003). However, a meta-analysis showed that parents do not differentiate between boys and girls with regard to parental control (Endendijk, Groeneveld, Bakermans-Kranenburg, & Mesman, 2016).

There are some unresolved issues in the literature on gender-differentiated parenting. First, it has been argued that fathers might be more involved with gender socialization than mothers (Eagly et al., 2000; Johnson,^1963^). Meta-analytically, there is indeed some evidence that fathers differentiate more between boys and girls than mothers (Lytton & Romney, 1991). However, this meta-analysis has been criticized for using too-broad categories of socialization behaviors, including few observational studies, and not weighting study results by sample size (Keenan & Shaw, 1997).

Second, almost all studies adopt between-family designs in which parenting in families with boys is compared with parenting in families with girls. It is, however, essential to examine whether boys and girls are also treated differently when they grow up in the same family (within-family comparison in families with both a boy and a girl) to take into account the possible influence of between-family differences (for example, differences in genes). A series of studies by McHale et al. has demonstrated more gender-differentiated parenting in families with mixed-gender siblings than in families with same-gender siblings in parenting domains of warmth, involvement, and knowledge about children’s activities (Crouter, Helms-Erikson, Updegraff, & McHale, 1999; McHale et al., 1999, 2000). In families with both a boy and a girl, opportunities for gendered comparisons are available which might emphasize differences between boys and girls and result in differential treatment of boys and girls (McHale et al., 1999).

Third, although gender-differentiated parenting has been labeled as an important factor influencing child behavior, very few studies have actually examined the link between gender-differentiated parenting and child behavior. One study showed that fathers attended more to submissive emotion in girls than in boys, whereas they attended more to disharmonious emotion in boys than in girls (Chaplin et al., 2005). Moreover, Chaplin et al. found that parental attention predicted later submissive emotions, and disharmonious emotions predicted later externalizing problems. However, they did not formally test for mediation, i.e., whether parent behavior mediates the association between child sex and child behavior. In another study, it was shown that mothers were more responsive to girls than to boys in a puzzle game, which was related to more happy, engaged, and relaxed behavior in girls than in boys during the puzzle task (Mandara, Murray, Telesford, Varner, & Richman, 2012). However, these associations were tested concurrently, and initial differences between boys’ and girls’ behavior may have confounded the results. Last, a recent study demonstrated that when fathers used physical control strategies more often with boys than with girls this was related to higher levels of aggression in boys than in girls a year later even when controlling for initial gender differences in aggression (Endendijk et al., 2017). Thus, gender-differentiated parenting indeed appears to be an important mechanism underlying children’s gender-typed behavior.

Fourth, it is difficult to disentangle child-sex effects on parenting or parental reactions from effects of gender-specific behavioral or temperamental differences. One study did control for gender differences in temperament and still found evidence for harsher treatment of boys than girls (Bezirganian & Cohen, 1992). Moreover, the classic experiments in which differential treatment was studied in response to the same baby that was dressed both as a boy and a girl also found evidence that parents treat boys and girls differently, regardless of their behavior (e.g., Culp, Cook, & Housley, 1983). These findings suggest that parents hold different attitudes about how to treat boys and girls. This does not preclude, however, that differential treatment of boys and girls occurs as a reaction to biologically predisposed gender differences in child behavior, or as a result of a combination of both parental attitudes and evocative effects of child behavior.

Some studies have examined possible moderators of the association between parental gender socialization and child gender-typed behavior. With regard to parent sex, it has been found that fathers’, but not mothers’, gender socialization was associated with later gender differences in social–emotional behavior (e.g., Chaplin et al., 2005; Endendijk et al., 2017). With regard to child sex, parents and other adults have been more concerned with socializing boys to show gender-typical behavior than they are with girls (Egan & Perry, 2001; Thomas & Blakemore, 2013). Moreover, girls have been found to be more resilient than boys to gender-conformity pressures from parents (Vantieghem & Van Houtte, 2015). Thus, the association between gender socialization and child gender-typed behavior is likely to be stronger in boys than in girls.

As will become evident in the section on cognitive theories of gender development, the influence of parental gender socialization on the child’s gender-typed behavior is likely to be at least partially mediated by the child’s cognitions about gender. In more recent advances of the social learning perspective on gender development (i.e., social cognitive theory of gender development; Bussey & Bandura, 1999), the importance of the child’s gender cognitions for the association between social learning and gender development is acknowledged. However, the mechanisms underlying the internalization of external factors, such as modeling or gender-differentiated parenting, into gender-related cognitive structures were not elaborated (Martin, Ruble, & Szkrybalo, 2002). Besides the mediation by the child’s gender cognitions, it seems likely that socialization pressures keep having a direct effect on child behavior, especially for younger children who are still developing their gender cognitions.

## Cognitive Approaches: The Role of Parent and Child Cognitions About Gender

### The Importance of Children’s Gender Cognitions for Gender Development

A central theme in cognitive perspectives on gender development is the idea that children are not passive recipients of all gender-related information and socialization from their environments, but instead play an active role in learning gender-typical behavior and gender-related cognitions (e.g., cognitive-developmental theory: Kohlberg, 1966; gender-schema theories: Bem, 1981, 1983; Martin & Halverson, 1981, 1987; developmental intergroup theory: Bigler & Liben, 2006). This is why the cognitive theories are often grouped together as “self-socialization” theories.

In his cognitive-developmental analysis, Kohlberg (1966) posited the importance of three cognitive stages for organizing gender development: gender identity, gender stability, and gender consistency. Gender identity refers to the ability to identify one’s own gender and later also other’s gender. Children need to have an awareness of their own gender and others’ gender to observe which behaviors are usually carried out by members of their own gender, to model the behavior of same-gender peers or adults, and to know which behaviors are considered appropriate for each gender. Gender stability, which generally develops a few years later, refers to understanding the fixed nature of gender over time. Last, gender consistency refers to the understanding that gender is invariant to changes in appearance or situations. Kohlberg ascribed children’s movement through the stages to the increasing complexity of children’s cognitive abilities during development. Even though Kohlberg’s ideas were innovative, the mechanisms underlying children’s self-socialization into gender roles were not clearly articulated. Also, Kohlberg was unclear about which stage of gender understanding was most important for organizing gender development. There is evidence that the highest stage of gender constancy, gender consistency, is associated with various aspects of gender development, but more and stronger associations are found between lower levels of gender constancy (gender stability, gender identity) and gender-typed preferences and behavior (for a review, see Martin et al., 2002). Also, in contrast to Kohlberg’s predictions, gender consistency has been found to be related to less rigid gender beliefs (Ruble et al., 2007).

In the 1970s and 1980s, several versions of gender-schema theory were developed independently from each other (GST: Bem, 1981, 1983; Martin & Halverson, 1981, 1987), taking into account the limitations of Kohlberg’s cognitive-developmental theory. These theories have in common that they tried to explain *how* children are active agents in gender socialization and development. Central to these theories are gender schemas—cognitive structures containing gender-related information. Gender schemas are dynamic in that they change in response to new experiences. These theories also assume that people do not passively absorb all information from the environment in their gender schemas, but instead selectively attend to the environment (e.g., own behavior, parents, siblings, extended family members, broader society and cultural environment) and actively construct schemas on the basis of the information that is attended to. Last, GSTs hypothesize that gender schemas influence the way we perceive gendered information and provide social standards that guide behavior. Gender schemas exert their influence for example by processes such as schema-directed memory (e.g., information about same-gender activities and behaviors). Gender schemas also provide a motivation to act in accordance with gender norms, because gender is related to the self-concept and because of the prescriptive nature of stereotypes (i.e., the affective component of the gender schema). From GSTs predictions, one could argue that there is a bidirectional association between gender-related behavior/experiences and gendered cognitions (i.e., gender schemas guiding own behavior and own/others’ behavior or experiences modifying gender schemas).

Evidence for the prediction that gender-related information gets incorporated in schemas is provided by studies examining gender categorization in children, as the ability to respond to males and females as members of separate categories is a fundamental aspect of building a gender schema. For example, 9–12-month-old infants are able to discriminate between men and women on the basis of hair length and clothing style, suggesting that schemas contain information about gender-typical hair and clothing (Leinbach & Fagot, 1993). Another study showed that gender discrimination might already be present in 3- to 4-month-old infants (Quinn, Yahr, Kuhn, Slater, & Pascalis, 2002). Around the age of 2, knowledge of gender-typical behavior, activities, and items is included in the gender schemas of children. This finding is demonstrated by a series of experimental studies in which infants were able to associate gender-typical toys and items with male and female faces (Eichstedt, Serbin, Poulin-Dubois, & Sen, 2002; Serbin, Poulin-Dubois, Colburne, Sen, & Eichstedt, 2001), and looked longer to stimuli showing males and females in activities that were inconsistent with gender stereotypes (Serbin, Poulin-Dubois, & Eichstedt, 2002). The influence of gender schemas on attention, perception, and memory is, for example, demonstrated by studies showing that 3- to 6-year-old children and adults have better memory for same-sex-typed familiar objects than for other-sex-typed familiar objects (Cherney & Ryalls, 1999). The same effect was found for unfamiliar objects (Bradbard & Endsley, 1983), which suggests that it is not the actual object, but the sex-typing of the object, that matters. Similarly, other studies found that children mistakenly remembered that schema-consistent information occurred more frequently than schema-inconsistent information, even though each was presented equally often (Susskind, 2003).

GSTs mainly focus on the influence of children’s own-gender schemas in relation to future behavior. However, its basic premises can also be applied to the intergenerational transmission of gendered ideas in societies and in families. For example, when gender is a salient issue in a family, due to the gender socialization behaviors of parents, this will encourage the continuation of gendered ideas in children, because they incorporate these early gender-related experiences in their own-gender schemas. Thus, GSTs propose an indirect pathway from parent behavior, to child gender cognitions, to child behavior, expanding on the direct pathway from parent-to-child behavior that is proposed by social learning theories. Also, GSTs expect a certain level of congruence between gender schemas and gendered behavior in both parents and children.

### Associations Between Parent and Child Gendered Cognitions and Behavior (Fig. 7)

According to GSTs, parents play an important role regarding the content of children’s gender schemas, because children are searching for gendered information in their environment, including their family, and form stereotypes based on their family members’ behavior. However, children also receive gender-related input from other agents such as peers, teachers, and the media (Dobbs, Arnold, & Doctoroff, 2004; Gooden & Gooden, 2001; McHale, Crouter, & Whiteman, 2003; Rose & Rudolph, 2006). Therefore, it is likely that the content of parents’ and child’s gender schemas will be similar but slightly different. Meta-analytically, there is evidence that parents’ gender schemas are related to their children’s gendered cognitions (i.e., parents with traditional gender schemas are likely to have children with traditional gender schemas as well), but the associations are small (Tenenbaum & Leaper, 2002).

**Fig. 7:**
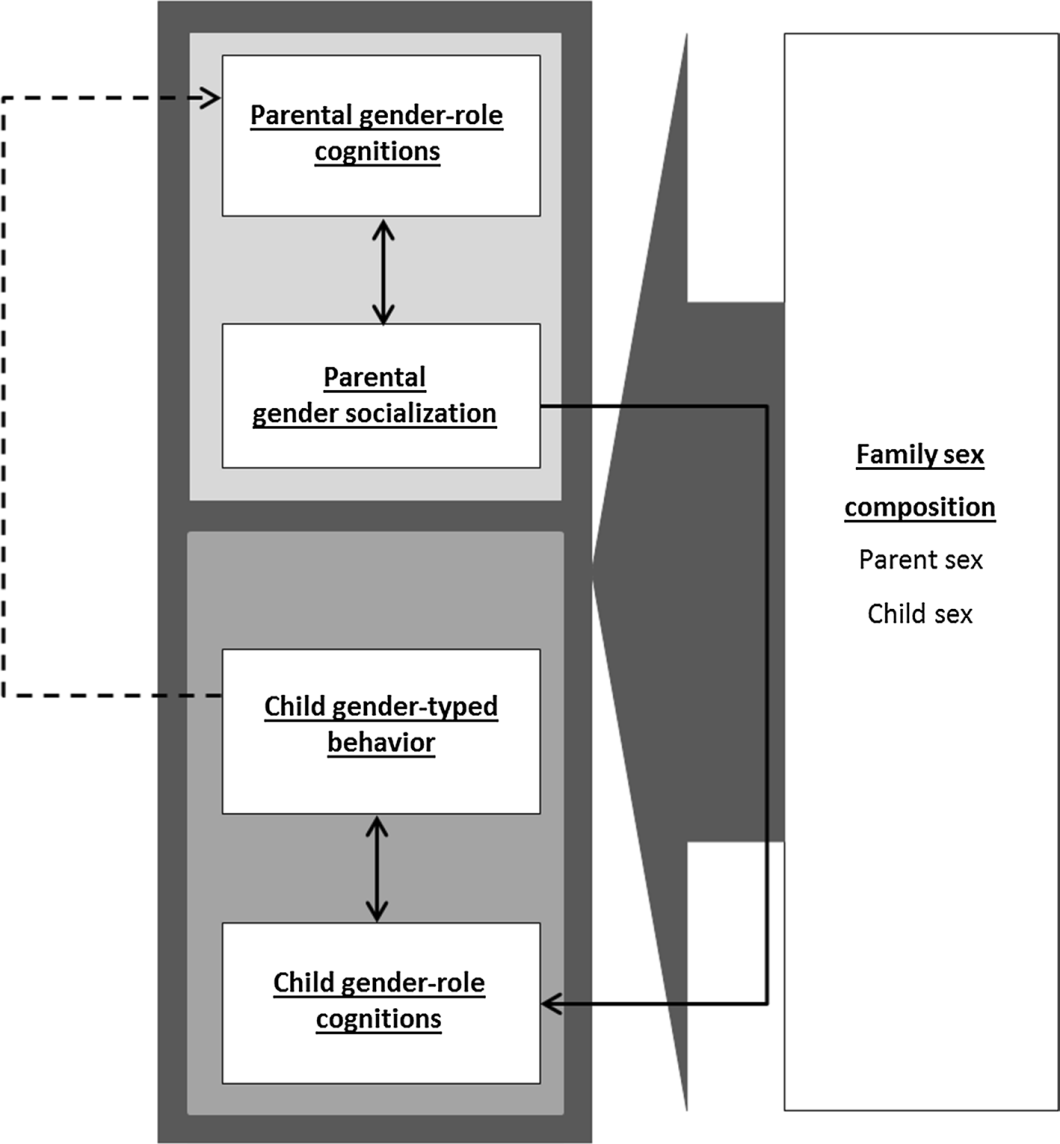
Associations between parent and child gendered cognitions and behavior

Although GSTs provide elegant explanations for the persistence of gender stereotypes and the intergenerational transmission of gendered ideas, the evidence for a link between parental gender stereotypes and actual parenting behavior in the family context is mixed (e.g., Fagot et al., 1992; Tenenbaum & Leaper, 2002), with most studies finding no significant associations. The evidence that is supporting the idea of a stereotype-behavior link in adults is often found with experimental studies or with highly structured tasks assessing cognitive processes like encoding or memory of, and attention to, gendered information (e.g., Frawley, 2008; Habibi & Khurana, 2012; Kee, Gregory-Domingue, Rice, & Tone, 2005; Kroneisen & Bell, 2013; Sherman, Stroessner, Conrey, & Azam, 2005). We know of only a few studies on gender-related parent–child conversation that have found meaningful associations between mothers’ gender stereotypes and the way they talk about gender with their children (Endendijk et al., 2014; Friedman et al., 2007; Gelman et al., 2004). For example, mothers with stronger gender stereotypes were more likely to make comments confirming gender stereotypes and to evaluate gender-role inconsistent behavior more negatively than mothers with more egalitarian gender-role stereotypes (Endendijk et al., 2014; Friedman et al., 2007). In addition, mothers’ traditional parenting style has been associated with more traditional gender-role stereotypes (Ex & Janssens, 1998).

The lack of a stereotype-behavior link for parents may be partly because parents’ stereotypes are often assessed explicitly (overtly expressed ideas about men and women), whereas for controversial subjects like gender and race, implicit stereotypes that operate largely outside conscious awareness may be better predictors of behavior than explicit self-reported stereotypes (Nosek, Banaji, & Greenwald, 2002). Self-report of gender stereotypes may be biased by social desirability and a lack of awareness of one’s own stereotypes (White & White, 2006). Another reason why few studies found a link between parental gender stereotypes and actual parenting behavior might be that parental gender stereotype measures often are not specifically measuring attitudes regarding the socialization of a parent’s own children (Blakemore & Hill, 2008).

In children, there is ample experimental evidence for the gender stereotype-behavior link (e.g., Bradbard & Endsley, 1983; Bradbard, Martin, Endsley, & Halverson, 1986; Davies, 1989; Martin, Eisenbud, & Rose, 1995; Montemayor, 1974). For example, in a classic experiment (Bradbard & Endsley, 1983), children played more with and had better memory for novel toys that were previously labeled by the experimenter for the child’s sex than for toys labeled for the other sex. Moreover, this preference for same-sex-labeled toys was even found in an experiment with novel toys varying in attractiveness in which demand characteristics were controlled for (i.e., one experimenter presented toys and another experimenter assessed toy preference; Martin et al., 1995). Also, children have been found to perform best at novel games labeled for their own sex and worst at games labeled for the other sex (Montemayor, 1974).

Only a few correlational studies on stereotype-behavior congruence in children have been conducted (Martin & Dinella, 2012). Children’s stereotypes about gender are also often assessed explicitly with questionnaires (Gender Attitude Scale for Children; Signorella & Liben, 1985; Children’s Occupations Activities and Traits scales; Liben & Bigler, 2002). One study showed high levels of congruence between self-reported gender stereotypes and preferences for stereotypical masculine or feminine activities of 7- to 12-year-old girls (Martin & Dinella, 2012). Another study focusing on adolescent girls’ academic achievement found that explicit egalitarian stereotypes about gender were related to more math and science motivation (Leaper, Farkas, & Brown, 2012). In addition, implicit math-gender stereotypes predicted academic achievement above and beyond explicit math-gender stereotypes for both boys and girls, and over and above enrollment preferences for girls (Steffens, Jelenec, & Noack, 2010). Last, children’s gender-typed beliefs about others’ playmate preferences were associated with children’s own playmate preferences, indicating that the more children thought other children preferred to play with same-sex playmates, the more they themselves preferred to play with same-sex playmates (Martin, Fabes, Evans, & Wyman, 1999). So, it appears that both children’s implicit and explicit stereotypes about gender are associated with child behavior.

With regard to the direction of effects in the association between gendered cognitions and behavior in parents and children, experimental studies clearly show that cognitions guide behavior (e.g., Bradbard & Endsley, 1983). Only a few studies provide evidence for the other direction in which behaviors change cognition, by showing that children’s own preferences for novel toys influence their ideas about whether a particular toy will be liked by girls or boys (Martin et al., 1995; Weisgram, 2016). According to GSTs, both directions are possible, but the idea of cognitions guiding behavior is most strongly proposed in earlier versions of GST. Only later the other direction in which personal experiences shape gender cognitions was considered to be important as well (Liben & Bigler, 2002; Martin et al., 1995).

Consistent evidence (albeit from a small number of studies) is present for the internalization of parents’ gender socialization practices into children’s gender cognitions (e.g., Barak, Feldman, & Noy, 1991; Ex & Janssens, 1998; Turner & Gervai, 1995). One study found that the more mothers employed a conformist parenting style (i.e., child has to comply with traditional norms and values) with their daughters, the more traditional the daughters’ gender-role stereotypes were (Ex & Janssens, 1998). In addition, mothers’ parenting style was largely influenced by their own-gender-role stereotypes, which suggests a pathway from parents’ gender-role stereotypes to parent behavior, and from parent behavior to children’s gender-role stereotypes. Another study that examined the traditionality of parents’ occupations, which can be seen as a reflection of their gender roles, showed that the traditionality of mothers’ occupations was related to children’s gender stereotypes (Barak et al., 1991). In addition, mothers and fathers who performed more nontraditional gender-role behaviors in the home had children with less strong gender stereotypes (Turner & Gervai, 1995).

To our knowledge, there are no studies conducted on the internalization of children’s gender-related behaviors into parents’ gender cognitions, although according to gender-schema theories (Bem, 1981, 1983; Martin & Halverson, 1981, 1987) and family system theories (Whitechurch & Constantine, 1993) it would be expected that children also influence parents’ stereotypes about gender. However, there are studies demonstrating that becoming a parent is associated with more traditional gender-role attitudes (e.g., Baxter, Buchler, Perales, & Western, 2015; Corrigall & Konrad, 2007; Katz-Wise, Priess, & Hyde, 2010), possibly because of the more traditional division of tasks between men and women after the arrival of a baby. Relatedly, child gender appears to be related to parents’ color preferences (Cohen, 2013). Having sons only increased fathers’ liking for blue, decreased mothers’ liking for blue, and marginally increased mothers’ liking for pink. This might indicate that children’s gender-typed color preferences affect parents’ gendered cognitions.

It is also important to mention that there are other-gender cognitions than gender stereotypes, such as intergroup attitudes and aspects of gender identity. However, these gender cognitions have been applied less frequently to gendered processes in the family context. Intergroup attitudes can be defined as the tendency to evaluate one’s own membership group (the in-group) more favorably than a nonmembership group (the out-group) (Tajfel & Turner, 1986). We know that both adults and children implicitly and explicitly evaluate their own gender positively and the other gender more negatively (Cvencek, Greenwald, & Meltzoff, 2016; Dunham, Baron, & Banaji, 2015; Halim, Ruble, Tamis-LeMonda, Shrout, & Amodio, 2016). Moreover, there is meta-analytic evidence that parent and child intergroup attitudes are related throughout childhood and adolescence (Degner & Dalege, 2013). Further, in adults there is ample evidence that in-group attitudes influence discriminative behavior to outgroup members (for a review, see Greenwald & Pettigrew, 2014). However, it is not known whether parents’ in-group attitudes influence the gender socialization practices with their children. In children one study showed that other-gender negativity predicts gender-biased behavior (Halim et al., 2016). Children who were more negative about the other gender rewarded a greater proportion of coins to the own gender than to the other gender and were sitting farther from an other-gender child than from an own-gender child.

Along with ability to identify one’s own gender, gender identity has been conceptualized in several other ways in the past decades (for a review, see Martin, Andrews, England, Zosuls, & Ruble, 2017). Examples are gender typicality (perceived compatibility with one’s gender group), gender centrality (how important gender is to one’s overall self-concept), contentedness with one’s gender, and felt pressure to conform to gender norms (Egan & Perry, 2001). Most recently, Martin et al. (2017) proposed a dual-identity approach in which gender identity involves both a connection to one’s own gender as well as to the other gender. In children and adolescents, gender identity aspects have been found to be associated with gender-typed attitudes (Leaper & Brown, 2008; Martin et al., 2017; Patterson, 2012) and gender-typed behavior, such as self-efficacy for gender-typed activities, male- and female-typed personality characteristics, liking of boys and girls, and sexual orientation (Egan & Perry, 2001). Studies in adults and parents have linked gender identity aspects to gender-role cognitions and gender-typed behaviors and outcomes, such as involvement in family roles, career success, and spatial abilities (for a review, see Wood & Eagly, 2015).

Last, there are yet other cognitions that might be relevant in a family process model, such as personal cognitions that influence social information processing (Crick & Dodge, 1994). Although these cognitions have hardly been applied to gendered family processes, they could provide a valuable direction for future research. According to the social information processing (SIP) model, a person’s behavior is the result of social information processes that are guided by social cognitions (Crick & Dodge, 1994). Several cognitions are relevant to the GFP model: attributions of the intentions of male and female family members in a situation (e.g., “women are naturally just more nurturing than men,” “boys’ disruptive behavior is innate”), expectations about the behavior of male and female family members in a situation (e.g., “boys that do not get their way will start acting out,” “fathers will be just as involved with family life as mothers”), appropriateness of a response (e.g., “boys should not play with dolls,” “mothers with small children should not work part-time”), gendered goals (e.g., “behave like a typical boy/girl,” “fostering gender-egalitarian values in my child”), and self-efficacy in one’s ability to enact a response (e.g., “I am able to balance work and family life,” “I can be assertive when my older brother wants to play with my toy”). Similar to GSTs, the SIP model assumes that cognitions and behaviors are interrelated between family members. For example, cognitions between family members influence their interactions. A family member’s behavior or previous interactions between family members can also influence other family members cognitions.

Several studies by the same research group have demonstrated the presence of gender differences in attributions of both parents and children, showing that boys’ risky misbehavior and injuries were attributed to inborn characteristics, whereas girls’ risky misbehavior was attributed to changeable situational factors (Morrongiello & Bradley, 1997; Morrongiello & Hogg, 2004; Morrongiello & Rennie, 1998). Consistent with these attributions, mothers would actively try to prevent risky misbehavior to daughters, but not to sons (Morrongiello & Hogg, 2004). Apparently, mothers’ gendered beliefs about the fixed/malleable nature of a person’s characteristics influence whether they tried to modify their children’s behavior (Dweck, 2000).

Very few studies have examined possible moderators of the stereotype-behavior link, even though it has been suggested that the influence of gender schemas on behavior is dependent on many factors within the child/parent and the environment (e.g., child’s developmental level, situational demands, salience, and accessibility of schemas; Martin et al., 2002). This association might be moderated by child or parent sex, because men/boys have been found to be more concerned with acting in accordance with their gender-role stereotypes than women/girls (Fischer & Arnold, 1994; Hort, Fagot, & Leinbach, 1990).

### Gender Differences in Gender-Role Cognitions (Fig. 7)

There are several studies on the differences between men and women in gender stereotypes, but the evidence is not conclusive. However, differences between studies might be primarily due to whether implicit or explicit stereotypes were assessed. When stereotypes are assessed explicitly, men display stronger gender stereotypes, whereas the level of implicit stereotypes is similar for men and women (Banaji & Greenwald, 1995; Rudman & Glick, 2001; Rudman & Kilianski, 2000) or somewhat stronger in women (Nosek et al., 2002; Osterhout, Bersick, & McLaughlin, 1997). Some studies do not find gender differences (Banaji & Greenwald, 1995; Swim, Aikin, Hall, & Hunter, 1995). A meta-analysis that focused specifically on parental gender stereotypes found that mothers hold less traditional attitudes about gender than fathers (Tenenbaum & Leaper, 2002), but most studies in this meta-analysis used explicit gender stereotype measures. More recent studies that also focused on parental stereotypes found similar results, with fathers reporting more traditional attitudes about gender than mothers (Blakemore & Hill, 2008) and mothers having more traditional implicit gender stereotypes (Endendijk et al., 2013). Several studies and a meta-analysis have demonstrated that the gender difference in implicit and explicit gender stereotypes is not yet present in preschool children (Endendijk et al., 2013; O’Brien et al., 2000; Signorella, Bigler, & Liben, 1993), but might emerge later in childhood (McHale et al., 1999). Knowledge of gender roles and gender stereotypes has been consistently found to emerge earlier in girls than in boys (Miller, Lurye, Zosuls, & Ruble, 2009; Poulin-Dubois, Serbin, Eichstedt, Sen, & Beissel, 2002; Serbin et al., 2001). With regard to in-group bias, both women and girls have been found to be more positive about their own gender than men and boys are about their gender (Dunham et al., 2015; Rudman & Goodwin, 2004).

Research comparing gender identity aspects between boys and girls has shown mixed results. On the one hand, boys have higher levels of own- versus other-gender similarity (Martin et al., 2017), gender typicality, gender contentedness, and felt pressure to behave in line with gender norms than girls have (Egan & Perry, 2001). On the other hand, some research has shown that girls, in fact, have higher levels of gender centrality than boys have (Turner & Brown, 2007; Verkuyten & Thijs, 2001). These differences may be due to age and cultural differences between the studies. Research in adults has shown that women score higher on gender centrality (Burn, Aboud, & Moyles, 2000) and identify more strongly with the gender in-group than men (Cadinu & Galdi, 2012), while no gender differences were found for gender typicality (Dambrun, Duarte, & Guimond, 2004).

## Family Sex Composition

Not all families are the same with regard to composition. A structural family characteristic that is especially relevant for gender-related family processes is the family sex composition, which consists of the sibling sex configuration (same-sex versus mixed-sex siblings) and the parent sex configuration (single-parent family, two-parent family, heterosexual, homosexual). Regarding parent sex configuration, data from the US Census Bureau have shown that the number of single-parent households increased in most Western countries from around 10% in the 1980s to around 30% in 2008 (US Census Bureau, 2012). However, there is considerable variation between countries; for example, the proportion of single-parent families in Japan was similar to that of the U.S. and UK in the early 1980s (US Census Bureau, 2012). Both the number of single-mother and single-father households increased. A small number of households consist of same-sex parents (e.g., USA: 0.1% male–male couples, 0.3%, female–female couples; Krivickas & Lofquist, 2011).

### Family Sex Constellation and Parent and Child Gendered Cognitions and Behaviors (Fig. 8)

In line with the family systems perspective (e.g., Whitechurch & Constantine, 1993), siblings play an important role in gender socialization, parent and child gender cognitions, and child gender-typed behavior (e.g., McHale et al., 1999; Rust et al., 2000; Stoneman, Brody, & MacKinnon, 1986). The GFP model thus includes a path from sibling sex configuration to parent and child gendered cognitions and behaviors. However, the results from the small number of studies conducted are mixed with regard to the direction of effects.

**Fig. 8:**
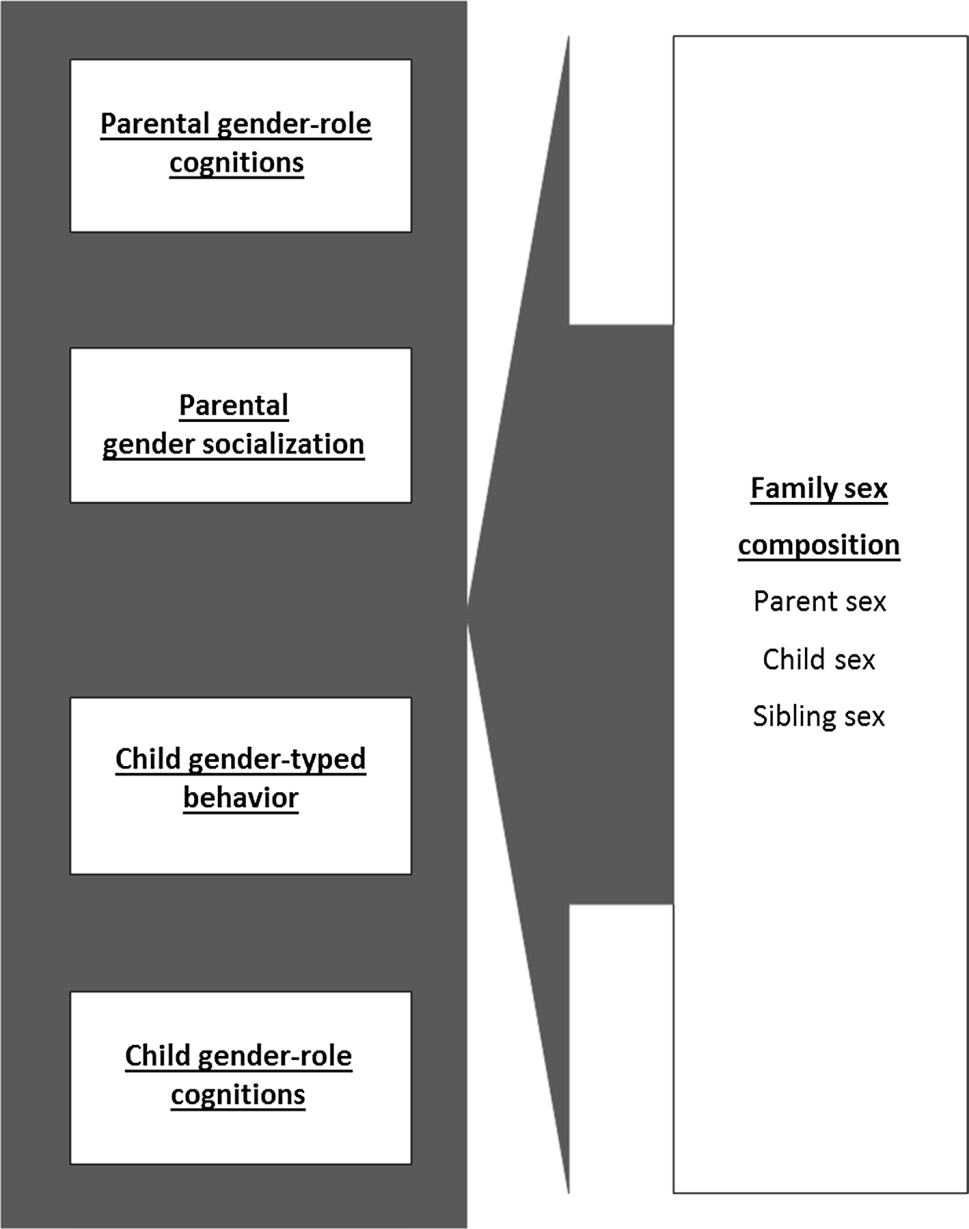
Family sex constellation and parent and child gendered cognitions and behaviors

First, there is evidence that siblings are an important source of observational learning and/or reinforcement of own-sex characteristics (e.g., Brim, 1958; McHale et al., 1999; Rust et al., 2000; Stoneman et al., 1986; Van der Pol et al., 2016). In families with a mixed-sex sibling configuration (e.g., boy–girl, girl–boy), the opposite-sex siblings reinforce opposite-sex behavior in each other. In families with same-sex siblings (e.g., girl–girl, boy–boy), the siblings are models for gender-typical behaviors, leading to an increase of gender-typical behavior in the siblings. In this case, the presence of an opposite-sex sibling may work as a gender neutralizer on the family environment (Brim, 1958; Rust et al., 2000). Recently, evidence has started to emerge that a mixed-sex sibling configuration also has a gender-neutralizing effect on parental behaviors and stereotypes, indicating that parental gender stereotypes and gender talk were more egalitarian in families with mixed-sex siblings compared to families with same-sex siblings (Endendijk et al., 2013, 2014).

Second, there is also evidence that siblings may serve as sources of social comparison or dis-identification in middle childhood (McHale et al., 1999). By showing very diverse behavioral patterns siblings can differentiate themselves from each other in the context of sibling rivalry for parental attention and fill a niche of their own in the family (Sulloway, 2010). Relatedly, in preadolescent and adolescent boys with gender identity disorder, sibling sex ratio of highly feminine males showed a significant excess of brothers (Blanchard, Zucker, Bradley, & Hume, 1995; Zucker et al., 1997). This could indicate that in all-boy families, boys might dis-identify with masculinity and identify more with femininity to fill a niche of their own. In families with a mixed-sex sibling configuration, parents have the opportunity for gender-differentiated parenting, which may provide a more gender stereotypical environment than families with same-sex siblings (e.g., McHale et al., 1999; McHale, Updegraff, Jackson-Newsom, Tucker, & Crouter, 2000). In this case, the presence of an opposite-sex sibling may work as a gender intensifier on the family environment, leading to an increase of gender-typical behavior in the siblings. Last, there are studies that do not find effects of sibling sex configuration on child behavior (Hauser & Kuo, 1998).

Thus, studies find mixed results with regard to the mixed-sex sibling configuration being a gender neutralizer or a gender intensifier in the family context. Recent research has shown that this might be due to the fact that mixed-sex settings only work as a gender intensifier on behavior when gender stereotypes are activated (Hirnstein, Andrews, & Hausman, 2014). However, it is also possible that social comparison and dis-identification processes become more important in later childhood and adolescence, because identity formation is an important goal at this age (Erikson, 1968). This could explain the gender-intensifying effects of having an opposite-sex sibling found in middle childhood in the McHale et al. (1999) study and not in the studies conducted in early childhood demonstrating gender-neutralizing effects (Endendijk et al., 2013, 2014; Rust et al., 2000; Van der Pol et al., 2016). Also, it is not yet known what the effect of family sex composition is on intergroup attitudes.

With regard to the pathway from parental gender configuration to gender-related cognitions and behavior, it is often thought that parents in nontraditional families (e.g., single-parent families, gay and lesbian parents,) hold less traditional stereotypes about gender and are less traditional in their behaviors than parents in traditional families. Biblarz and Stacey (2010) examined these hypotheses in an extensive review of the literature. They concluded that single-sex parenting (i.e., single-parent, gay and lesbian parents) appears to foster more androgynous parenting practices in both mothers and fathers. Families with single-sex parents do not only employ different socialization practices, they are also models for nontraditional gender roles to their children. Single parents’ behavior indeed is often less traditional, because these parents have to fulfill both gender roles of economic provider and caretaker. The same is true for gay and lesbian parents, who are more likely to share the roles of caretaker and economic provider (Solomon, Rothblum, & Balsam, 2005; Stacey & Biblarz, 2001).

It seems reasonable to expect that children in these nontraditional families (gay and lesbian parents, single parents) would also hold less traditional stereotypes about gender and show less gender-typical behavior. However, meta-analytically there are no significant differences between children with heterosexual or homosexual parents, or between children from single-mother families or families with a mother and a father, with regard to sexual orientation, gender identity, satisfaction with life, and cognitive and moral development (Allen & Burrell, 1997; Fedewa, Black, & Ahn, 2015). Unexpectedly, children of homosexual parents scored higher on traditional gender-role behaviors than children of heterosexual parents, but the combined effect size was small and showed publication bias (Fedewa et al., 2015). Also, many different gender-role behavior measures were combined in this meta-analysis. Thus, these results need to be interpreted with caution.

## Future Directions and Conclusion

Our review of the literature on gender in the family context and the new GFP model resulting from the review highlight the interplay of biological, social, and cognitive factors in gender-related family processes. The review also reveals important gaps in the literature that need to be addressed in future research (see Table 2). In all three domains (i.e., biology, socialization, cognition) of research on gender development, there is a clear need for more experimental and longitudinal studies including both mothers and fathers and preferably starting before birth and continuing into puberty. Before birth, hormones in amniotic fluid, maternal blood, or umbilical cord blood can be measured (Hines et al., 2015; van de Beek, Thijssen, Cohen-Kettenis, van Goozen, & Buitelaar, 2004) to examine the influence of prenatal T on gender development in typically developing children. Previous research using these methods showed promising results (Constantinescu & Hines, 2012). However, T measured in maternal blood or umbilical cord blood might not be an accurate proxy of the level of T the fetus is exposed to, because T is actively metabolized in the placenta (Hollier, Keelan, Hickey, Maybery, & Whitehouse, 2014). Thus, the results of studies using these measures need to be compared with natural experiments using atypical samples such as girls with CAH, who we know are exposed to high levels of prenatal T. In addition, both mothers’ and fathers’ hormonal profiles can be assessed before actual parenthood, or manipulated experimentally (see Bos et al., 2012), to investigate the direction of effects regarding the association between parental testosterone levels and parenting behavior. After birth, parental T levels can be related to both quantitative (e.g., parental involvement) and qualitative aspects of parenting behavior (e.g., sensitivity, emotional availability) as well as more specific gender socialization practices of parents. It is important to use observational next to self-report measures of parents’ gender socialization practices, since gender socialization practices in the family context are generally very subtle and often happen outside parents’ conscious awareness (Culp et al., 1983).

**Table 2:** Directions for future research

Domain	Gaps/limitations in previous research	Direction for future research
Overall	Need for studies examining interplay between biology (e.g., T levels), gender socialization, gender-role cognitions, and gender-typed behavior in typically and atypically developing children Direction of effects unclear Few father studies	Longitudinal studies and/or experimental studies Including mothers and fathers Starting before birth Start assessing parental T levels before parenthood, or manipulate parents’ T levels experimentally Cross-lagged design (both parent and child gendered behavior and cognitions assessed at multiple time points) Mixed methods: biological measures, observations, (self-report) questionnaires, computer tasks assessing implicit gender stereotypes Examine differences and similarities in gendered family processes in typically developing children versus children with disorders of sex development, or children with gender dysphoria
Biological	Ethical/methodological difficulties with experimentally manipulating T levels Few studies on prenatal T in normative samples More attention to puberty	Study the effects of T in adolescents or adults with gender identity disorder who receive hormonal treatment to suppress puberty or to enhance cross-gender secondary sex characteristics A paradigm that can be used for examining effects on parenting is the Leiden Infant Simulator Sensitivity Assessment (LISSA; Voorthuis et al., 2013) that makes use of an infant simulator (RealCare Baby II-Plus; Realityworks, Eau Claire, WI, USA). Prenatal T assessments in amniotic fluid/maternal blood/umbilical cord blood Attention to validation of these measures; comparison of associations between prenatal T and gender-typed behavior with studies using atypical samples such as girls with CAH, who are exposed to high levels of prenatal T Continue longitudinal studies into puberty with attention to activational effects of T
Social	Few studies with within-family designs Focus on dyads in a family	Compare gender-differentiated parenting *within* families to gender-differentiated parenting *between* families Are socialization differences between boys and girls also found when they grow up in the same family, thus when the same parents socialize both a boy and a girl? Examine triadic or total-family interactions to directly examine the effect of family sex configuration on family interaction patterns
Cognitive	Need for studies examining: (a) link between parental cognitions and behavior (b) Associations between biological factors and gender cognitions	Include implicit measures or neuroscientific measures of gender stereotypes Focus on multiple aspects of gendered cognitions and their interrelations within and between family members Examine family sex composition in relation to parent and child intergroup attitudes and gender identity

Longitudinal studies should employ a cross-lagged design (both parent and child behavior assessed at multiple time points) in which the complex issue of child-to-parent and parent-to-child reciprocal effects with regard to gender-differentiated parenting could be examined appropriately. With such studies, it is also possible to empirically test the widely held assumption that parental gender socialization practices have an important impact on the development of gender-typed behavior (Archer & Lloyd, 2002). However, the focus should not only be on examining the influence on gender differences between boys and girls but also on individual differences within boys’ and girls’ gender development (McHale et al., 2003). When the assessments are extended into puberty, it is possible to examine the effects of biological, social, and cognitive changes associated with puberty on gender-related family processes. This can shed light on whether puberty can be considered as a period of “gender-intensification” (Hill & Lynch, 1983) in which boys and girls become increasingly different as a result of the convergence of biological, social, and cognitive changes. Previous research on this regard has shown mixed results (e.g., Priess, Lindberg, & Hyde, 2009), possibly because of historical changes in gender equality, patterns of gender socialization, and gender development. Future studies should also compare gendered family processes in families with typically developing children versus families with children with disorders of sex development (e.g., CAH), or children with gender identity disorder, to test whether the propositions of the GFP model hold in general or are specific to certain populations.

A specific direction for future research in the biological domain of gender development arises from the fact that studies in this domain are hampered by the difficulty (ethical and methodological) to conduct experiments in which T levels are externally manipulated. An opportunity to study the effects of T on parenting experimentally is provided by adolescents or adults with gender identity disorder who receive hormonal treatment to suppress puberty or to enhance cross-gender secondary sex characteristics. As the majority of gender-dysphoric adults do not have children of their own, a paradigm that can be used for this is the Leiden Infant Simulator Sensitivity Assessment (LISSA; Voorthuis et al., 2013) that makes use of an infant simulator (RealCare Baby II-Plus; Realityworks, Eau Claire, WI, USA). It might be interesting to examine the parenting quality (e.g., sensitivity) of these individuals with the simulator before and after the hormonal treatment or to compare parenting quality of individuals who have received the hormonal treatment with matched controls who have not yet received this treatment.

A specific direction for future research for studies with a social approach toward gender development arises from the fact that studies in the social domain often adopt a between-family design to examine differences in parenting boys and girls. An important limitation of this approach is that differences in parenting practices toward boys and girls do not necessarily reflect a gender difference, but can also be caused by confounding factors that differ between families. It is of vital importance to compare gender-differentiated parenting within families to gender-differentiated parenting between families to account for such factors. The crucial question to be addressed in the within-family design is whether socialization differences between boys and girls are also found when they grow up in the same family (when the same parents socialize both a boy and a girl). Only then can we be more sure that systematic variations in parenting boys and girls cannot be ascribed to other family variables. More within-family studies are needed to disentangle the effect of child sex on parenting practices from between-family effects. However, these studies need to be designed carefully with attention to age-matching or controlling for birth order effects.

In studying gender-related processes in the family context, future researchers should move beyond investigating children’s dyadic interactions with parents or other members in the nuclear or extended family context. Triadic interactions are now widely used to investigate family dynamics, and it has been consistently found that fathers’ and mothers’ behaviors with their child differ when observed in dyads versus triads (e.g., McHale, Fivaz-Depeursinge, Dickstein, Robertson, & Daley, 2008; Sacrano de Mendonça, Cossette, Strayer, & Gravel, 2011). For example, in a dyadic interaction setting there were no significant differences between mothers’ and fathers’ interactional synchrony with their child, whereas in a triadic setting fathers displayed less interactional synchrony with their child than mothers (Sacrano de Mendonça et al., 2011). It might be interesting to examine whether mothers’ and fathers’ gender socialization practices are also different in triadic compared to dyadic interactions. It may even be possible to extend the triadic interaction paradigm to quadratic interactions to directly examine the effect of family sex configuration on family interaction patterns.

In studies with a cognitive approach toward gender development, the evidence for the link between parents’ gender cognitions (stereotypes, intergroup attitudes, gender identity aspects) and the actual gender socialization behaviors toward their children is scarce. The same is true for studies examining child and parent biological factors, such as testosterone, on gender cognitions. More studies (experimental or longitudinal) should investigate associations between gender cognitions and behavior in parents and between parent/child biological factors and gender cognitions. These studies should incorporate implicit measures or neuroscientific measures (e.g., EEG, fMRI) of gender cognitions, since for controversial subjects like gender or race implicit stereotypes appear to be better predictors of behavior (Nosek et al., 2002). Future studies should also examine which gender-related experiences in the family context influence gender stereotypes in parents, since little is known about the internalization of these experiences into gender concepts. Further, it might be interesting to examine whether intergroup attitudes of family members are different between families with different family sex compositions. Last, it is important to examine interrelations between gendered cognitions and their combined effects on parent and child gendered behavior, as there is evidence that gender identity, gender stereotypes, and self-concept interact with each other (Baron, Schmader, Cvencek, & Meltzoff, 2014), and how they interact might influence gendered behavior.

To conclude, our review of the literature has shown that gender is indeed an important organizer of family processes. Based on the review, pathways between gender-related biological, social, and cognitive factors at both the parent and child level have been identified and integrated in a new comprehensive explanatory model of gendered family processes. In addition, the review demonstrated that to date much is still unclear about the mechanisms underlying gender-related processes within the family context, partly due to methodological limitations of previous research. Therefore, future studies should take into account the complexity of gendered family processes, by using advanced research designs, methods, and analytic approaches, studying interactions between biological, social, and cognitive factors in relation to gender-related family processes and child gender development. These studies can draw on the GFP model for pertinent research questions and theoretical grounding of their hypotheses so that we can fully understand how gender influences family processes.
